# Sirt3 deficiency induced down regulation of insulin degrading enzyme in comorbid Alzheimer’s disease with metabolic syndrome

**DOI:** 10.1038/s41598-022-23652-5

**Published:** 2022-11-17

**Authors:** Alpna Tyagi, Musa Musa, Wladimir Labeikovsky, Subbiah Pugazhenthi

**Affiliations:** 1 Rocky Mountain Regional VA Medical Center, Aurora, CO USA; 2 Department of Medicine, University of Colorado-Anschutz Medical Campus, Aurora, CO 80045 USA; 3 Department of Education and Research, Strauss Health Sciences Library, University of Colorado Anschutz Medical Campus, Aurora, CO 80045 USA

**Keywords:** Neuroscience, Neurology, Pathogenesis

## Abstract

SIRT3 deacetylates mitochondrial proteins, thereby enhancing their function. We have previously demonstrated that Sirt3 gene deletion leads to brain mitochondrial dysfunction and neuroinflammation. We also reported that silencing of Sirt3 gene in APP/PS1 mice results in exacerbation of insulin resistance, neuroinflammation and β amyloid plaque deposition. To further understand how metabolic syndrome and amyloid pathology interact, we performed RNA-seq analysis of the brain samples of APP/PS1/Sirt3^-/-^ mice. Gene expression patterns were modulated in metabolic and inflammatory pathways by Sirt3 gene deletion, amyloid pathology, and the combination. Following Sirt3 gene deletion, a key finding was the decreased expression of insulin-degrading enzyme (IDE), an enzyme that regulates the levels of insulin and Aβ peptides. Western diet feeding of Sirt3^-/-^ and APP/PS1 mice resulted in decrease of IDE protein, parallel to Sirt3 downregulation. Conversely, activation of SIRT3 by nicotinamide riboside in vivo and in vitro resulted in IDE upregulation. SIRT3 activation in vivo also increased the levels of neprilysin, another Aβ degrading enzyme and decreased the levels of BACE1 which generates Aβ peptide suggesting SIRT3’s role in amyloid plaque reduction. Our findings provide a plausible mechanism linking metabolic syndrome and amyloid pathology. SIRT3 may be a potential therapeutic target to treat AD.

## Introduction

Metabolic syndrome (MetS) is the precondition for diabetes, obesity, and cardiovascular disease^1^. It consists of a cluster of risk factors including hypertension, insulin resistance, hypertriglyceridemia, low HDL-cholesterol, and abdominal obesity. MetS is highly prevalent in developed countries with one in three adults having at least three of these risk factors^2^. A long-term study examined the connection between MetS and cognitive impairment in 1,759 elderly women and observed that patients with all five components of MetS were three to four times more likely to develop cognitive dysfunction than those without risk factors^3^. Specifically, insulin resistance, determined by the Homeostatic Model Assessment for insulin Resistance (HOMA-IR) index, was significantly linked with increased cognitive dysfunction. Another study observed that the individuals with worsening of MetS status over a five-year period had a greater chance of developing dementia^4^. MetS has been also shown to be a risk factor for Alzheimer’s disease (AD), the most common form of dementia. Tsai et al. examined the associations between individual components of MetS and the risk for AD and found a significant linear relationship between decrease in cognitive scores with increased number of MetS risk factors^5^. Hyperglycemia showed maximum effects on cognitive decline followed by hypertension in this study. The link between MetS and cognitive dysfunction have been further supported by studies in both genetic and diet-induced animal models for MetS^6–9^. However, the CNS effects of MetS at the cellular and molecular levels are not clearly understood.

MetS is caused by genetic risk factors and lifestyle changes^10^. One of the genetic risk factors is the downregulation of Sirt3 gene^11^. A single point mutation in human Sirt3 leads to reduced activity and MetS^12^. SIRT3 belongs to sirtuin family consisting of seven members that play critical roles in longevity and inflammation^10,13–15^. SIRT3 plays a central role in mitochondrial function. It deacetylates and activates key mitochondrial proteins. Targets of SIRT3 include metabolic enzymes, antioxidant enzyme SOD2, respiratory chain complexes and transcriptional coactivator PGC-1α, needed for mitochondrial biogenesis^16^. Sirt3^-/-^ mice fed on a calorie-rich diet is a well-known model for MetS that combines genetic risk factor with life-style changes^8^. Studies in these models have focused mainly on changes in peripheral tissues. Not much of studies have been done on the CNS effects of MetS. We examined the brain samples of Sirt3^-/-^ mice, fed on western diet and observed downregulation of metabolic enzymes, peripheral and central insulin resistance, mitochondrial dysfunction and inflammasome formation leading to neuroinflammation^8^. These changes in the brain are all potential triggering factors for Alzheimer’s pathogenesis. Therefore, to determine if metabolic syndrome in mid-life is a risk factor for AD, we generated a comorbid AD mouse model by deleting Sirt3 gene globally in APP/PS1 mice, an Alzheimer’s transgenic mouse model with amyloid pathology. We observed significant exacerbation of peripheral and central insulin resistance and elevation of neuroinflammatory markers in APP/PS1/Sirt3^-/-^ compared to APP/PS1 mice. The deposition of β amyloid plaques increased in terms of number and size because of Sirt3 gene deletion. Microglial proliferation and activation were significantly more in the comorbid AD brain. This exaggerated phenotype is not a surprise, because when you superimpose two pathologies, namely, MetS and amyloid plaque deposition, additive effects are expected. Our main goal of generating this mouse model is to determine how MetS and amyloid pathology interact with each other at the molecular level so that we can identify potential converging pathways. Therefore, the objective of this study is to perform RNA-seq analysis of the brain samples and profile the gene expression patterns following Sirt3 gene deletion in wild type as well as in APP/PS1 mice.

## Results

### RNA-seq analysis reveals differential gene expression patterns following Sirt3 gene deletion

**Figure 1:**
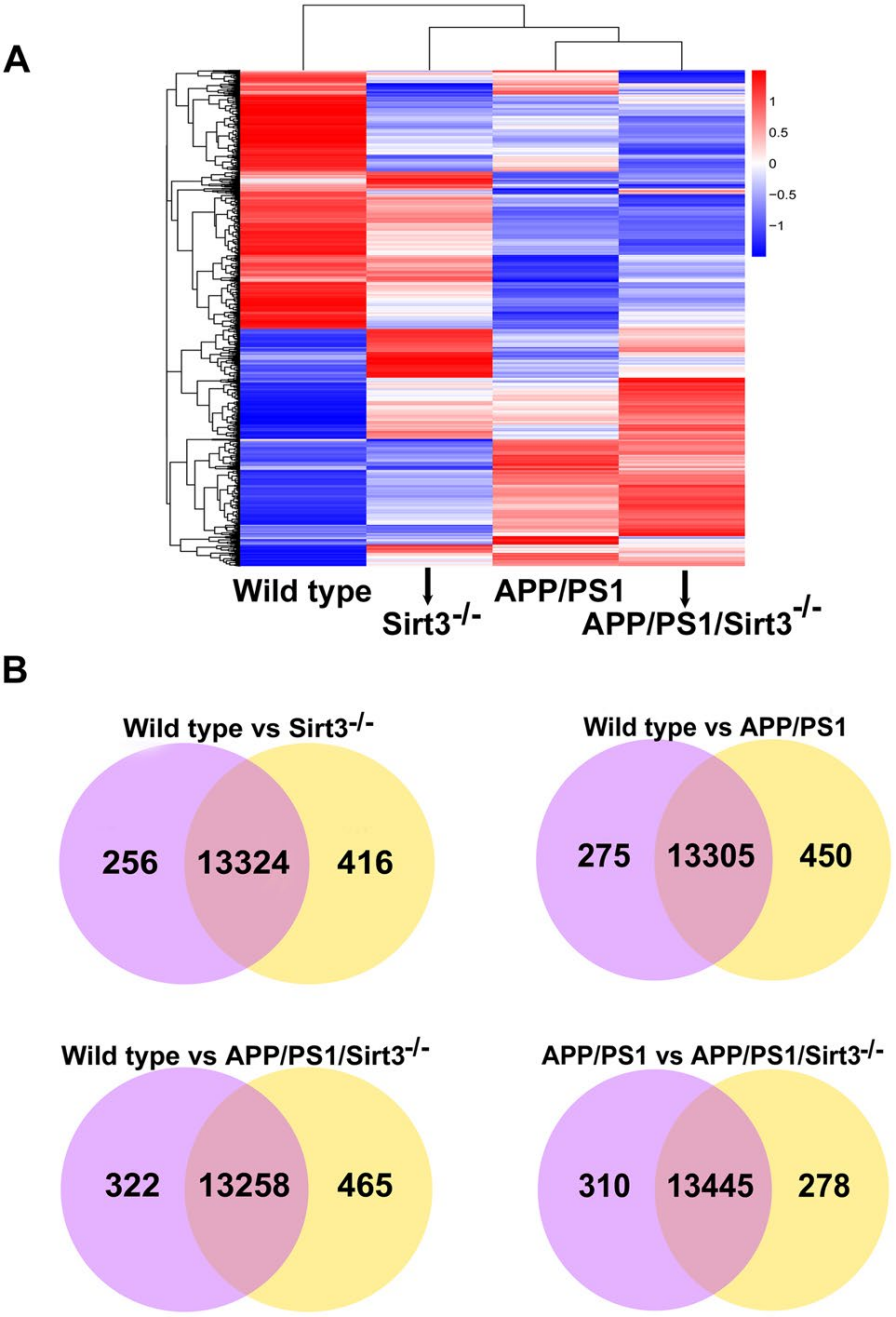
Heat map and Venn diagrams. Total RNA was isolated from the brain samples of 8 mo-old female mice (4 each) in wild type, Sirt3^-/-^, APP/PS1 and APP/PS1/Sirt3^-/-^ mice. After quality control, mRNA was purified, sheared into short fragments of ~ 200 bases and used as templates for cDNA synthesis, followed by library preparation. The cDNA libraries were sequenced (25 million reads), using Illumina NexSeq550 platform. RNA-seq reads were mapped to the mouse genome, using TopHat software. The heat map of cluster analysis (**A**) shows significant changes in the gene expression patterns between the groups. Unique and overlapping gene expressions between groups are shown by Venn diagrams (**B**).

**Figure 2:**
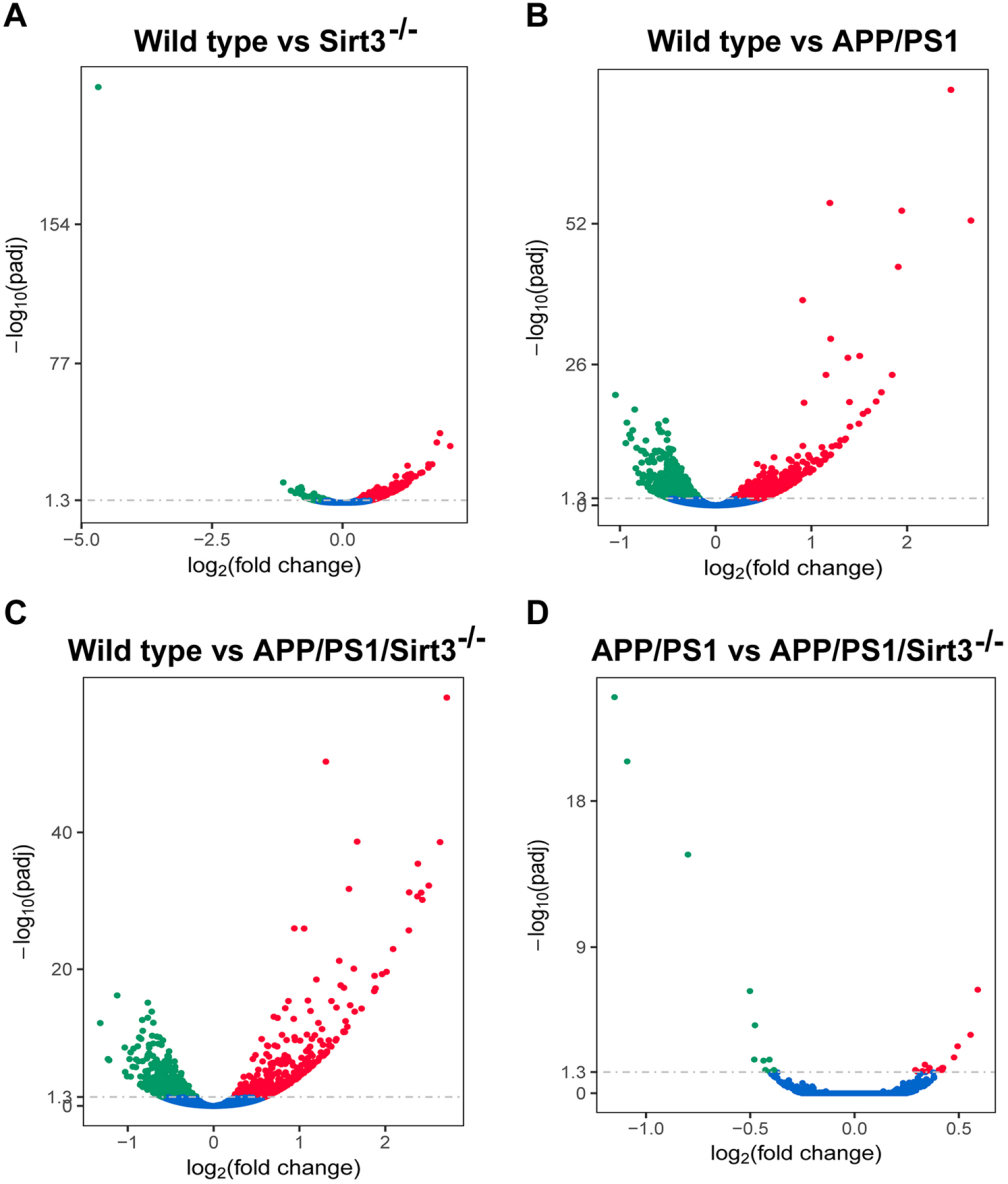
Volcano plots. Following RNA-seq analysis with the brain samples of 4 four groups of mice as in Fig. 1, differential gene expression patterns were analyzed and volcano plots showing upregulated (red) and downregulated (green) genes were drawn. Comparisons were made to determine the effects of Sirt3 gene deletion (**A**), amyloid plaque deposition (**B**) and the combination (**C**). Comparisons were also made to determine the effects of Sirt3 gene deletion in APP/PS1 mice (**D**).

Overall, the heat map of cluster analysis shows significant changes in the gene expression patterns between the 4 groups, namely wild type, Sirt3^-/-^, APP/PS1 and APP/PS1/Sirt3^-/-^, at 8 months of age (Fig. 1A). Pearson correlation between samples in each group were > 0.98. Unique and overlapping gene expressions between groups are shown in Venn diagrams (Fig. 1B). Four comparisons were made to determine the effects of MetS, amyloid pathology and the combination. The same comparisons were also made with Volcano plots showing upregulated (red) and downregulated (green) genes (Fig. 2). Sirt3 gene deletion, amyloid plaque deposition and the combination altered the gene expression patterns significantly. Specifically, comparison between APP/PS1 and APP/PS1/Sirt3^-/-^ mouse brain samples shows the effects of MetS when superimposed with amyloid pathology. The altered gene expression pattern suggests molecular interactions between MetS and amyloid pathology.

### Key hits among the altered genes following Sirt3 gene deletion

**Figure 3:**
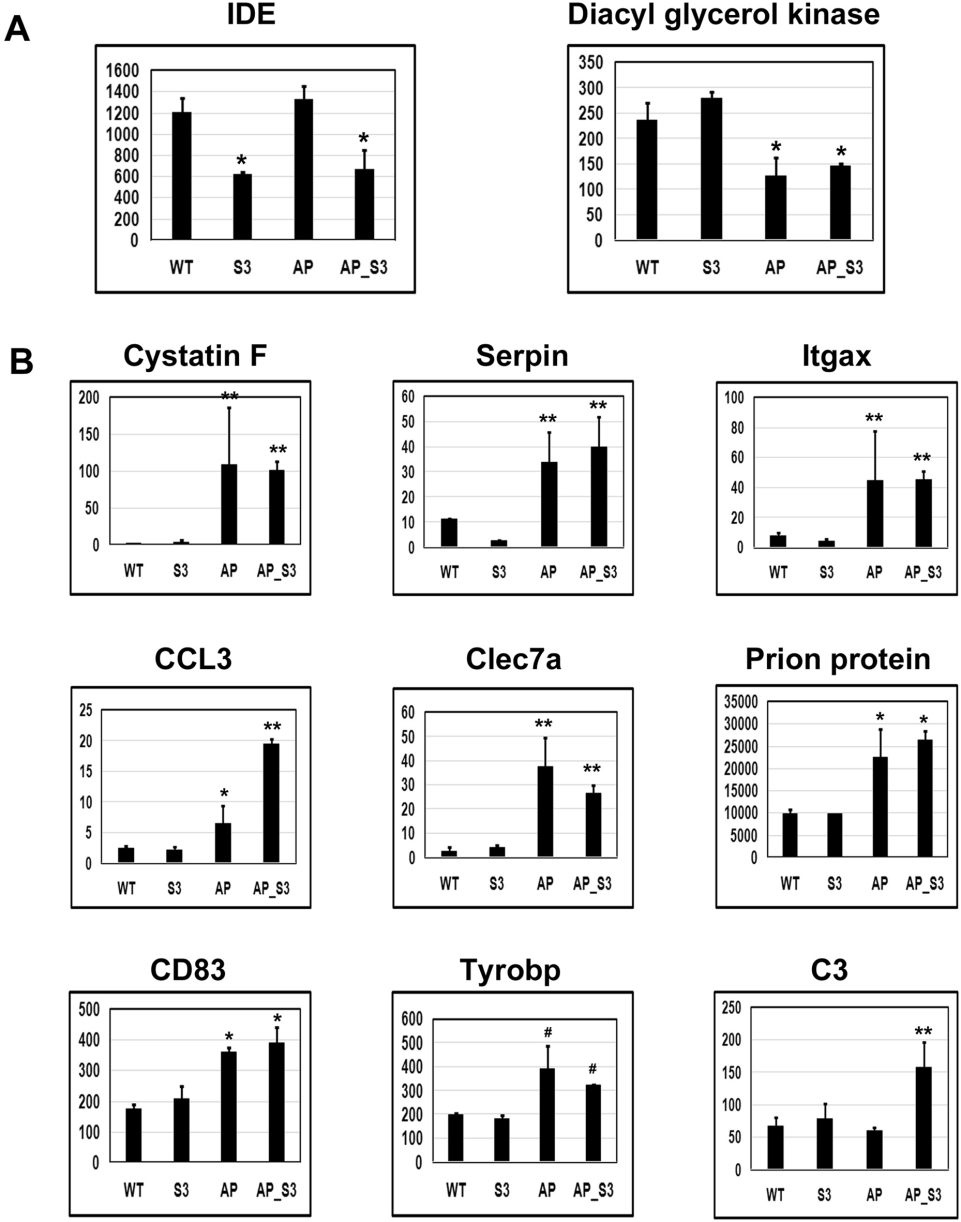
Altered gene expression by Sirt3 gene deletion and amyloid plaque deposition. RNA-seq reads for selected downregulated (**A**) and upregulated (**B**) genes are presented for wild type (**WT**), Sirt3^-/-^ (**S3**), APP/PS1 (**AP**) and APP/PS1/Sirt3^-/-^ (**AP_S3**) mouse brain samples. Data are presented as mean ± SE of 3 samples. ^#^*P* < 0.05; *P < 0.01 and **P < 0.001 compared to wild type mice.

Selected genes with modulated expression are presented in Fig. 3. We had previously demonstrated that Sirt3 gene deletion-induced mitochondrial injury leads to inflammasome formation in the brain^8^. Amyloid pathology is also known to induce neuroinflammation^17^. As expected, many among the upregulated genes were inflammatory mediators including serpin, CCL3, Clec7a, Tyrobp and C3. Cystatin F was induced by ~ 50 fold by amyloid deposition. The levels of chemokine CCL3 and itgax, also known as CD11C were induced by ten and five-fold respectively (Fig. 3B). CD83, a surface marker for matured dendritic cells and Tyrobp were induced modestly. These inflammatory markers did not change further when superimposed with MetS. However, the levels of complement protein C3 were elevated significantly more in comorbid AD mouse brain, compared to APP/PS1 mice, suggesting the role of Sirt3 downregulation. Decreases (*P* < 0.01) in the expression of diacyl glycerol kinase were observed in AD and comorbid AD mouse brain. This enzyme plays significant roles in brain function including cognition^18^. A key hit among the downregulated genes because of Sirt3 gene deletion was insulin-degrading enzyme (IDE) which plays a regulatory role in insulin turnover as well as degradation of Aβ peptide. The decreases were ~ 50% (*P* < 0.01) in Sirt3^-/-^ and APP/PS1/Sirt3^-/-^ mouse brain samples (Fig. 3A).

### Pathway analysis of differentially expressed genes

**Figure 4:**
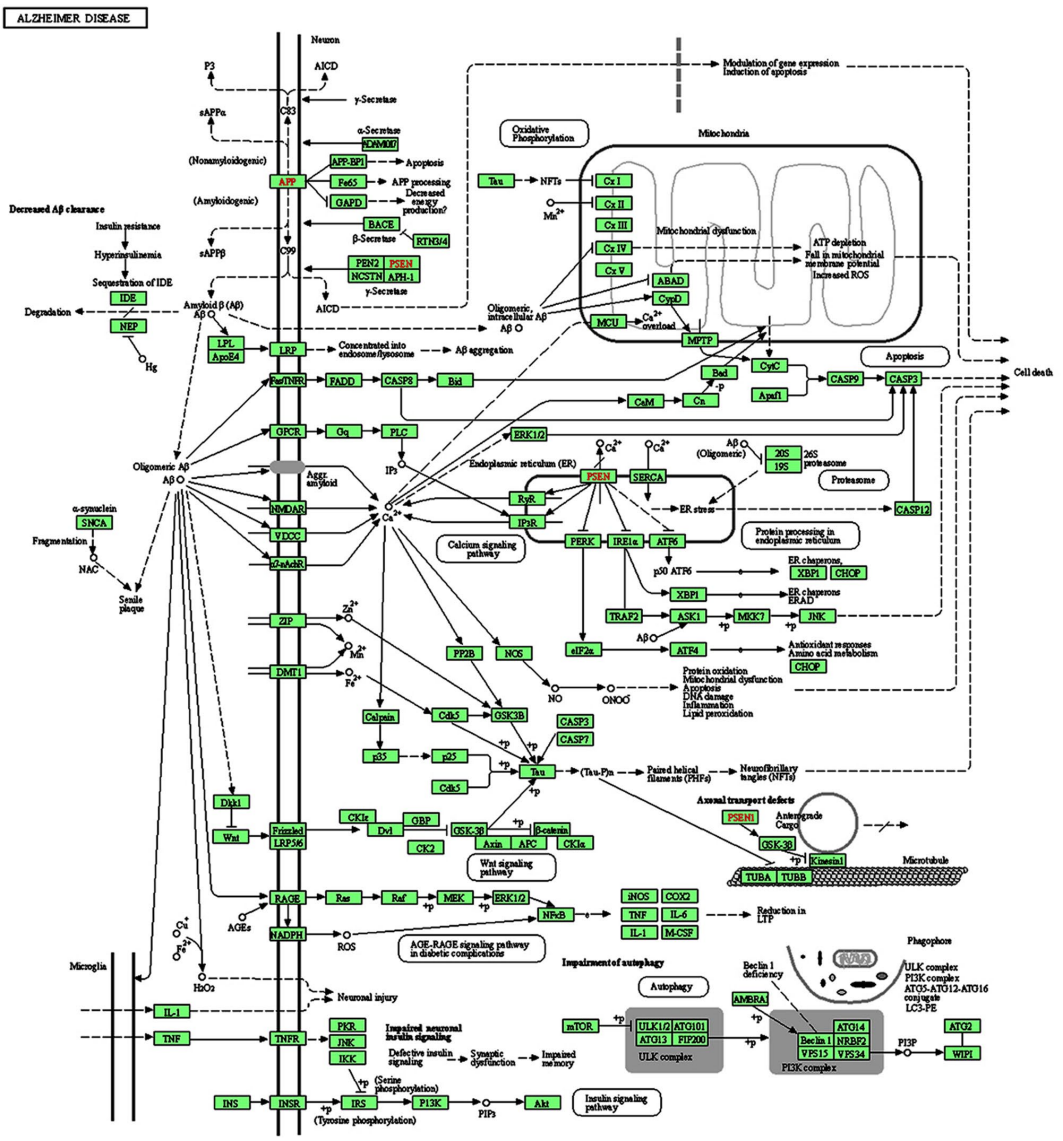
Pathway analysis: The differentially expressed genes (DEGs, 25,000) from wild type, Sirt3^-/-^, APP/PS1 and APP/PS1/Sirt3^-/-^ mouse brain samples were uploaded into the DAVID database (https://david.ncifcrf.gov/) for KEGG-GO pathway enrichment analysis in APP/PS1/Sirt3^-/-^ (comorbid-AD) mice. Enrichment analysis of KEGG revealed that DEGs were primarily involved in metabolic, inflammation, Alzheimer disease (AD) and insulin signaling pathways. A representative AD cascade pathway was obtained from KEGG Pathway Maps^19^**.** For the functional annotation of differentially expressed genes, the web-based DAVID Bioinformatics Resources v 6.8 tool was used (https://david.ncifcrf.gov).

GO and KEGG pathway analyses were performed with the differentially expressed genes. The web-based DAVID Bioinformatics Resources v 6.8 tool was employed (https://david.ncifcrf.gov). Overall, MetS-mediated gene expression changes in AD background were primarily involved in metabolic, inflammation, AD and insulin signaling pathways (Fig. 4). Out of 25,000 genes, 1599 genes in metabolic pathways, ~ 1000 genes in inflammatory pathways (292 genes in cytokine-cytokine receptor interaction; 292 genes in MAPK signaling pathway; 192 genes in chemokine signaling pathway; 168 genes in JAK-STAT signaling pathway; 137 genes in oxidative phosphorylation; 105 genes in NF-κB signaling pathway), 381 genes in amyloid pathway and 139 genes in insulin signaling pathway were significantly dysregulated in APP/PS1/Sirt3^-/-^ mice (Table 1). These results are in line with our previous findings that metabolic and inflammatory pathways are dysregulated in comorbid-AD mouse model (APP/PS1/Sirt3^-/-^)^7^.

**Table 1:** KEGG-GO pathways of altered genes in APP/PS1/Sirt3^-/-^ (comorbid-AD) mice.

Pathways	Gene count	*P* value
Metabolic pathways	1599	9.9E-23
**Inflammatory pathways**		
Cytokine-cytokine receptor interaction	292	1.5E-5
MAPK signaling pathway	292	5.4E-4
Chemokine signaling pathway	192	1.2E-3
JAK-STAT signaling pathway	168	3.3E-3
Oxidative phosphorylation	137	5.0E-2
NF-κB signaling pathway	105	4.4E-2
Alzheimer disease	381	1.5E-5
Insulin signaling pathway	139	5.0E-2

The significance of enrichment (*P* value) is shown in the table.

### Effects of calorie overload on the regulation of SIRT3 and IDE

**Figure 5:**
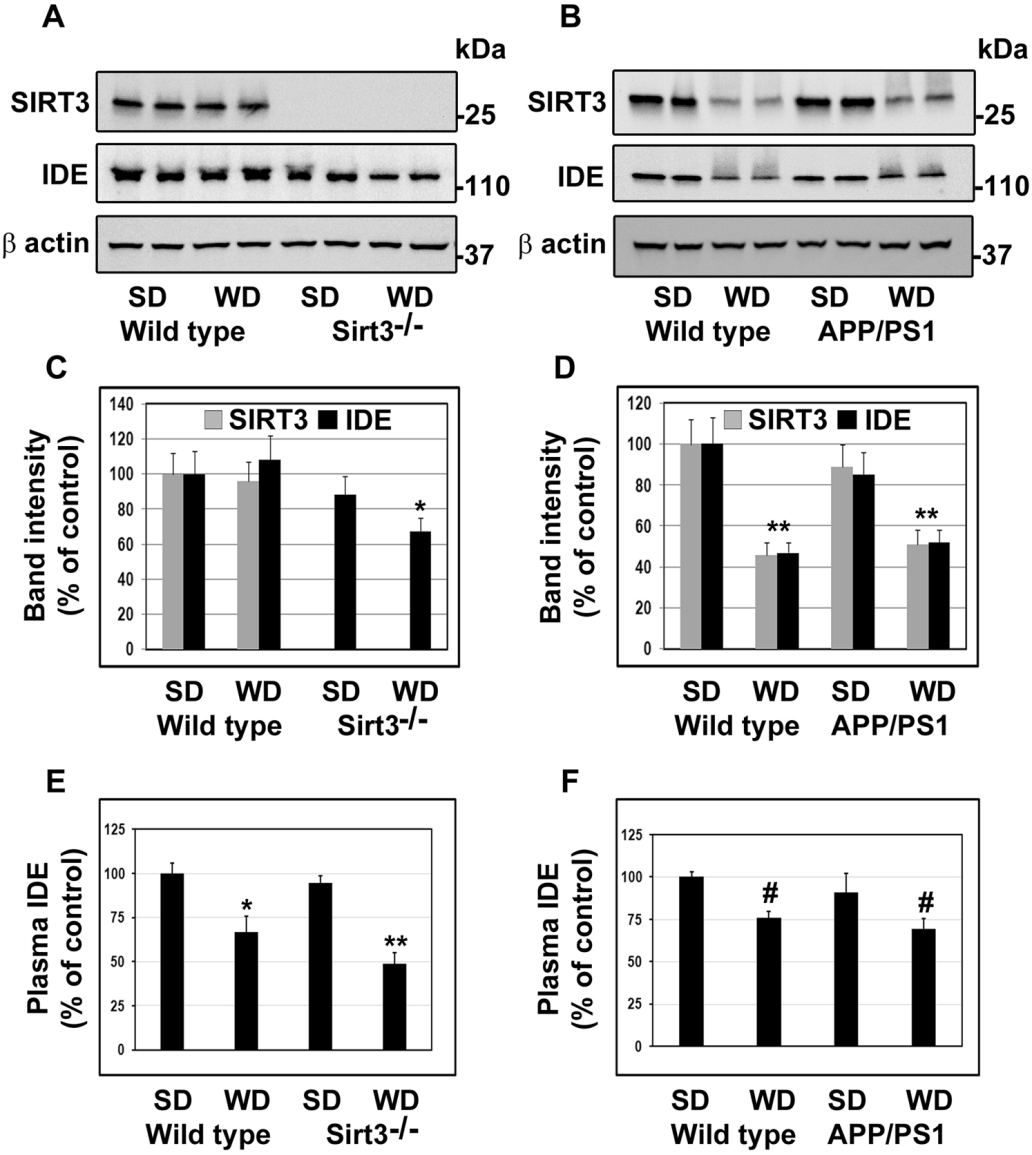
Effects of calorie overload on the levels of SIRT3 and IDE. Two-month-old male wild type and Sirt3^-/-^ mice were fed a standard diet (SD) or western diet (WD) for 4 months (**A**,**C** and **E**). 6 weeks-old Wild type (WT) and APP/PS1 male mice were fed on standard diet (SD) or energy-rich western diet (WD) for 7 months (**B,D** and **F**). Mouse cortical samples from the four groups of mice were collected for the Western blot analysis of SIRT3 and IDE (**A**,**B**). Representative cropped images are presented for clarity and conciseness while the composite color images of full blots are presented in Supplementary Figure. The band intensities were determined by scanning, corrected for β actin levels, and expressed as percent of control. (**C**,**D**). Plasma was separated from the collected blood samples for the assay of IDE by ELISA (**E**,**F**). Data are expressed as mean of ± SE (n = 6) for each group. #, *P* < 0.05; *, *P* < 0.01; **, *P* < 0.001 versus WT mice on standard diet.

Calorie overload is generally used to induce MetS in animal models. We had previously examined the effects of feeding western diet enriched in saturated fat and simple sugars in Sirt3^-/-^^8^ and APP/PS1^15^ mice. These mice were characterized by hyperinsulinemia, hypertriglyceridemia and weight gain. Calorie overload also led to decrease of SIRT3 levels in the brain. To determine if a decrease in SIRT3 causes parallel change in the IDE levels, Western blot analysis of the brain samples was performed. In Sirt3^-/-^ mouse brain samples, IDE levels decreased, especially following western diet feeding by 33% (*P* < 0.01; Fig. 5A,C). Plasma IDE also decreased by 48% (*P* < 0.001) in these mice (Fig. 5E). Western diet feeding in APP/PS1 mice resulted in ~ 50% decreases in the levels of SIRT3 as well as IDE (*P* < 0.001; Fig. 5B,D). However, the plasma levels of IDE in these mice decreased modestly (24%; *P* < 0.05; Fig. 5F).

### In vivo effects of SIRT3 activation by NR

**Figure 6:**
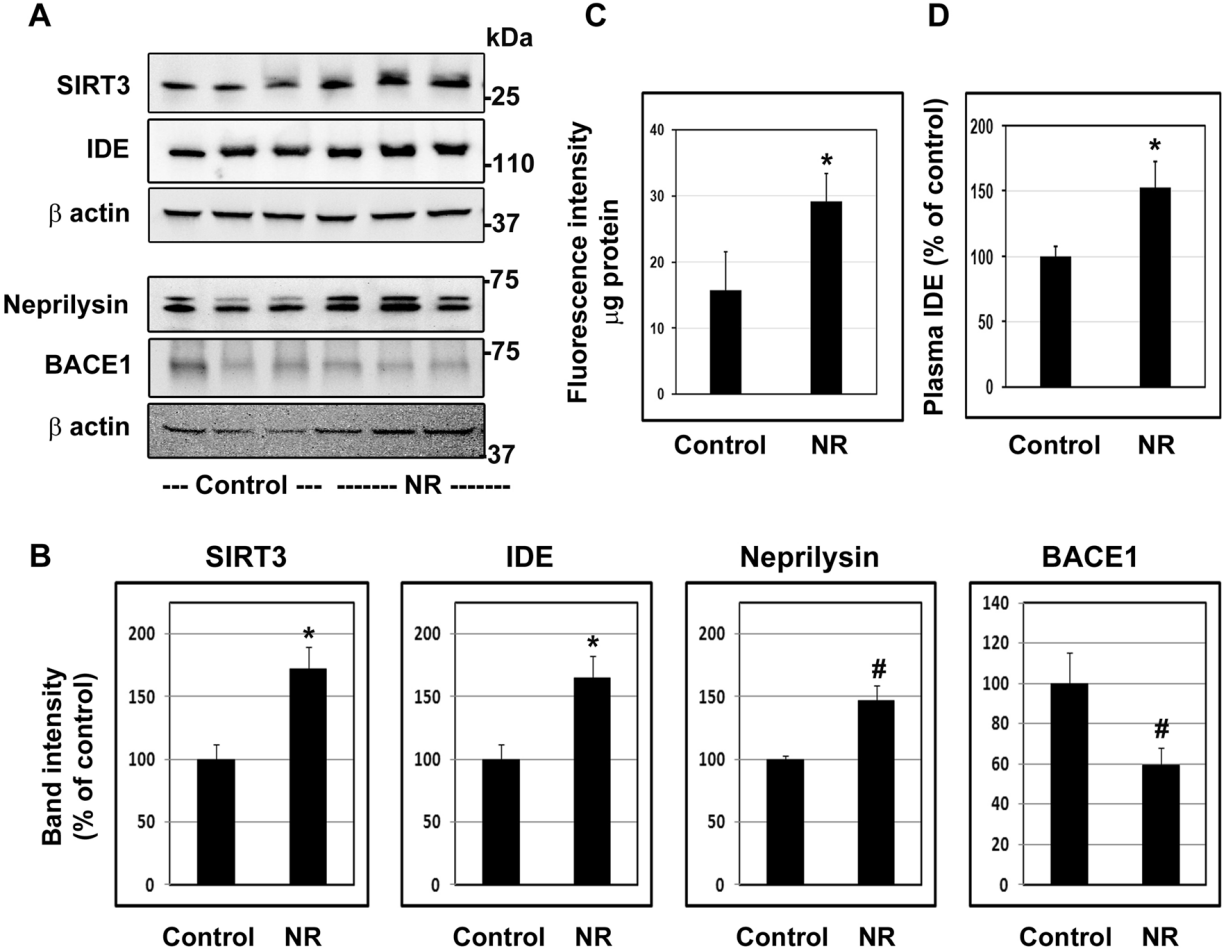
In vivo effects of SIRT3 activation by NR. Seven-month-old C57BL/6 male mice (WT, 6/group) were fed regular chow or diet supplemented with NR (0.4% W/W) for 3 months. The mice were sacrificed, and cortical samples and blood were collected. Western blot analysis was performed to determine the levels of SIRT3, IDE, neprilysin and BACE1 (**A**). Representative cropped images are presented for clarity and conciseness while the composite color images of full blots are presented in Supplementary Figure. The band intensities were measured by scanning and corrected for the levels of β actin. (**B**). IDE activity was measured with the cortical samples by a fluorometric assay. The activities were expressed as fluorescence units per µg protein (**C**). Plasma was separated from the blood samples and IDE levels were determined by ELISA (**D**). Mean ± SE of 6 samples are presented. #, *P* < 0.05; *, *P* < 0.01; **, *P* < 0.001 compared to Control untreated mice. NR, nicotinamide riboside.

SIRT3 is activated by nicotinamide adenine dinucleotide (NAD+). To increase the cellular content of NAD+ , its precursor, nicotinamide riboside (NR) is generally administered. In the current study, treatment of wild type mice with NR resulted in the upregulation of Aβ degrading enzymes namely IDE (65%; *P* < 0.01), neprilysin (47%; *P* < 0.05). In addition, the levels of BACE1 which generates Aβ decreased significantly (46%; *P* < 0.05; Fig. 6A,B). Increases in SIRT3 levels (72%; *P* < 0.01) were also observed, suggesting autoregulation of SIRT3 expression following its activation. IDE activity measured by a fluorometric assay showed an increase of 84% parallel to the protein levels (*P* < 0.01; Fig. 6C). The plasma levels of IDE were elevated (52%; *P* < 0.01) following NR treatment (Fig. 6D). Taken together, these findings support the role of SIRT3 in reducing amyloid plaque deposition through multiple pathways.

### Effects of SIRT3 activation by NR in cultured mouse microglial cell line

**Figure 7:**
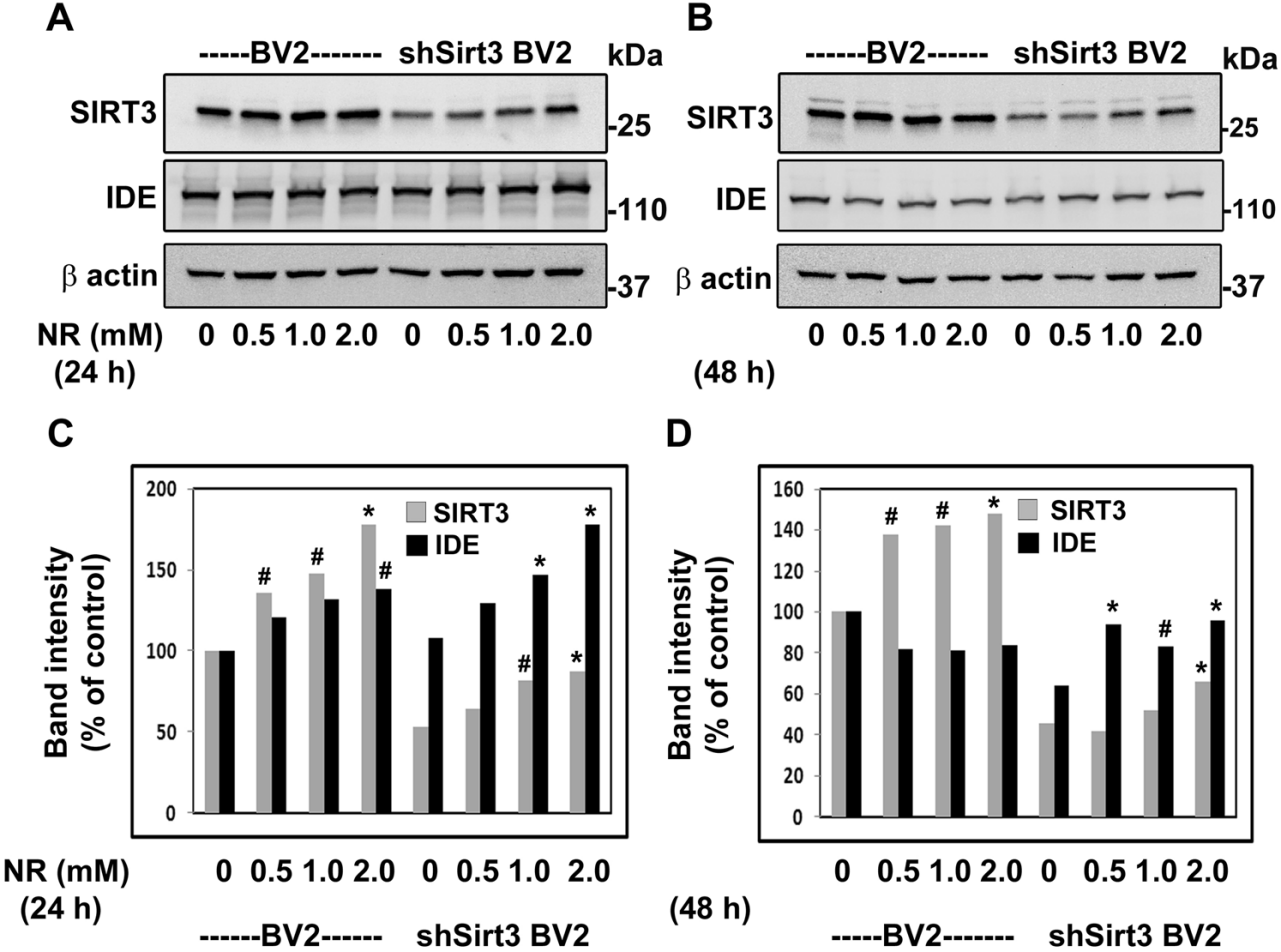
Effects of SIRT3 activation by NR in cultured mouse microglial cell line: BV2 and Sirt3-silenced shSirt3BV2 cells were cultured in the absence and presence of increasing concentrations of nicotinamide riboside (NR) for 24 h (**A**,**C**) and 48 h (**B**,**D**). The cell lysates were prepared and immunoblotted for SIRT3 and IDE (**A**,**B**). Representative cropped images are presented for clarity and conciseness while the composite color images of full blots are presented in Supplementary Figure. The blots were quantitated by scanning and corrected for the levels of β actin (**C**,**D**). Mean ± SE of results from 3 independent experiments are presented. *#, P* < 0.05; *, *P* < 0.01; **, *P* < 0.001 versus untreated control in respective cell lines, compared to Control untreated mice.

Next, we tested the effects of SIRT3 activation in BV2 cells, a mouse microglial cell line. We also used a BV2 cell line in which SIRT3 expression is significantly reduced by shRNA approach. We observed dose-dependent upregulation of SIRT3 levels in both cell lines. Activation of SIRT3 resulted in the upregulation of SIRT3 itself as like in vivo study. For example, there was a 78% increase of SIRT3 levels in BV2 cells after treatment with 2 mM NR for 24 h. In shSirt3 BV2 cells the basal SIRT3 levels were 47% less whereas after 24 h treatment with 2 mM NR, SIRT3 levels increased by 64% (Fig. 7A,C). Similarly, there were significant (*P* < 0.05—*P* < 0.01) increases in IDE levels, suggesting Sirt3-mediated induction. This effect was more pronounced in shSirt3 BV2 cells with an increase of 64% (*P* < 0.01). NR action on SIRT3 and IDE was more at 24 h compared to 48 h. At 48, there was no induction of SIRT3 by NR and IDE induction was modest (Fig. 7B,D). It appears that after initial induction, these proteins expression reached at their steady state level.

## Discussion

Sirt3^-/-^ mouse is a well-studied model to investigate the effects of MetS^8,11,20,21^. Previous studies with this model have focused mainly on the peripheral tissues. We had reported for the first time that MetS syndrome causes decreased mitochondrial function and neuroinflammation in the brain^8^. Subsequently, we generated a novel mouse model for comorbid AD, by silencing Sirt3 gene in APP/PS1 mice, an Alzheimer’s mouse model with amyloid pathology^7^. We observed in these AD mice, exacerbation of plaque deposition, neuroinflammation and astrogliosis when superimposed with MetS. To further understand how amyloid pathology and MetS interact at the molecular level, we performed RNA-seq analysis of the brain samples in the current study. We observed modulation of genes in the metabolic and inflammatory pathways because of Sirt3 gene deletion, suggesting that MetS can interact with amyloid pathology in the brain. A key finding of this study is the decreased expression of IDE following Sirt3 gene deletion. The relationship between SIRT3 and IDE was further tested in mice by feeding western diet or by treatment with NR, an activator of Sirt3. Diet-induced MetS decreased SIRT3 and IDE in wild type as well as in Alzheimer’s background. Activation of SIRT3 by treatment with NR in vivo and in cultured BV2 cells resulted in induction of IDE. IDE is involved in insulin homeostasis and its downregulation leads to insulin resistance^22^. IDE is also one of the enzymes that degrades Aβ peptide^23,24^. Similarly, the levels of neprilysin were elevated whereas BACE1 levels decreased following NR-mediated SIRT3 activation in vivo. Therefore, our findings suggest that Sirt3 deficiency as in MetS can exacerbate amyloid deposition in addition to interaction with AD through insulin resistance, mitochondrial dysfunction and neuroinflammation.

AD is characterized by extracellular amyloid plaques and intracellular neurofibrillary tangles^25,26^. There are also additional factors including mitochondrial dysfunction, insulin resistance, metabolic dysregulation and neuroinflammation that contribute to cognitive decline in AD^10,27,28^. Because of the complex nature of this disease, more than 100 clinical trials targeting individual pathology have failed. Recent studies have shown that the pure form of Alzheimer’s disease is very rare, and it often coexists with other comorbidities in the aging population. A ground-breaking report that reviewed the Nun Study (NS) and Honolulu-Asia Aging Study (HAAS) concluded that the total burden of brain lesions is the main cause of cognitive decline in AD^29^. Many of these brain lesions are caused by diseases including obesity, diabetes, hypertension and cardiovascular diseases. The precondition for these comorbidities is known as MetS. If we examine the timelines of comorbidities and AD, MetS, the precondition for comorbidities is generally seen in mid-life. In the case of AD, clinical dementia is preceded by mild cognitive impairment (MCI) and before that, there is a long prodromal cellular phase which is also in mid-life. Therefore, MetS may provide a fertile ground in the brain on which the progression of Alzheimer’s pathology may be facilitated in susceptible individuals^10^. Global silencing of Sirt3 gene is one of the approaches to induce MetS in animal models. Therefore, we generated APP/PS1/Sirt3^-/-^ mouse as a model for comorbid AD with amyloid pathology and MetS. We had previously reported additive effects of these two pathologies^7^. The current study was undertaken to determine the altered gene expression patterns in the brain.

Amyloid pathology and Sirt3 gene deletion resulted in significant changes in gene expression in the brain. These genes were predominantly in the inflammatory pathway (Fig. 3). The chemokine CCL3 and complement protein C3 were induced significantly more in APP/PS1/Sirt3^-/-^ mouse brain, compared to APP/PS1 mice, suggesting exacerbation of neuroinflammation in comorbid AD*.* However, the induction of cystatin F, CD83, Itgax and prion protein were comparable in APP/PS1 and APP/PS1/Sirt3^-/-^ mouse brain sample. Cystatin F is a protease inhibitor selectively expressed in immune cells^30^ and CD83 a surface marker for matured dendritic cells^31^. Itgax is also known as CD11C^32^. CD11C + microglia are known to be elevated in AD. Cellular production of neurotoxic Aβ is regulated by prion protein^33^. The upregulation of these genes seems to play significant roles in Aβ driven Alzheimer’s pathogenesis. Induction of some of the other genes may be a protective response to amyloid pathology. For example, Tyrobp is an adapter protein for TREM2 and it is considered as neuroprotective^34^, Serpin3k plays an antioxidant role^35^ and Clec7a is a phagocytic marker in microglia^36^.

A key finding of this RNA-seq analysis was the downregulation of IDE expression following Sirt3 gene deletion. As our aim was to test the link between MetS and AD, we decided to investigate IDE further because of its roles in insulin homeostasis as well as in Aβ degradation. IDE is a 110 kDa zinc metallo-endopeptidase that is known to degrade bioactive peptides including insulin, glucagon, amylin and Aβ peptide^37,38^. It is a ubiquitously expressed enzyme with liver being its major source. It is known to be localized in multiple intracellular compartments including mitochondria and endosomes. Leissring et al. have reported that an isoform of IDE, generated by translation from an upstream site, adds a 41 amino acid mitochondrial targeting sequence^39^. Inositol phosphates (InsPs) and phosphatdidyliinositol phosphates are potent activators of IDE. They localize IDE to endosomes where its substrates are present^40^. The IDE-C and IDE-N domains connected by a hinge switch between open and closed conformations, allowing intermediate size peptides to enter the pocket and get processed. This study also identified the endogenous IDE with the mitochondrial fraction. In addition to its proteolytic activity, the non-catalytic function of IDE has been also reported. For example, IDE interacts with the glycoprotein (GE) from varicella zoster virus (VZV)^41^. While this interaction contributes to skin virulence, it is not needed for VZV infection of T cells in vivo^42^. The other non-proteolytic functions of IDE include interaction with glucocorticoid receptors and α-synuclein^43,44^. SIRT4, another member of the sirtuin family has been shown to interact with IDE and this interaction appears to play a role in insulin secretion by β cells^45^. It will be of interest to see if SIRT3 plays a similar role in future studies.

Aβ peptides released by the proteolytic action of α and β secretases on the transmembrane amyloid precursor protein (AAP), polymerize to generate Aβ oligomers, protofibrils and amyloid plaques^46^. Excessive generations of Aβ oligomers and Aβ fibrils cause neurotoxicity in the AD brain. Along with neprilysin, IDE plays a significant role in degrading and clearing Aβ peptides. Therefore, conditions in which IDE is downregulated, exacerbated Aβ generation can be expected. Increased accumulation of endogenous Aβ has been reported in IDE^-/-^ mouse brain^24^. These mice were also characterized by insulin resistance. Genetic variations in IDE have been shown to be associated with risk for late-onset AD^47–50^. Li et al. have reported decreases in the levels of IDE in the brain samples of a mixed model AD mice with diabetes^51^. Brain IDE activity has been shown to decrease with age and in the early AD^52^. Transgenic overexpression of IDE enhances the proteolysis of beta-amyloid and decrease plaque formation^53^. IDE expression decreases in a mouse model with AD and type 2 diabetes^51^. IDE expression was restored following treatment with rosiglitazone and AICAR, activators of PPARɣ and AMPK.

The upstream pathways leading to IDE induction have been reported previously. IDE promoter contains Nrf1 binding site^54^. Nrf1 homodimerizes and functions as a transcription factor that activates the expression of some key metabolic genes. It is also required for mitochondrial respiration, and mitochondrial DNA transcription and replication. Nrf1, together with Nrf2, mediates the coordination between nuclear and mitochondrial genomes. Nrf1 in turn is induced by PGC-1α. Because Sirt3 induces the expression of PGC-1α, Sirt3 may play an indirect role in the transcription of IDE through a pathway involving PGC-1α and Nrf1. Acute exercise increases the expression of IDE in the liver and skeletal muscle^55^. Similarly exercise also increases SIRT3 expression^56^. Although SIRT3 may not be directly involved in IDE expression at the transcriptional level, there seems to be an indirect link between the two through PGC-1α and Nrf1. Further detailed studies are needed to determine SIRT3’s role in IDE expression. Significantly, SIRT3 activation by NR also resulted in the elevation of neprilysin, another Aβ degrading enzyme and significant decrease in the levels of BACE1, known as β secretase which generates Aβ peptides (Fig. 6A,B). Honokiol, another activator of SIRT3 has been also shown to decrease BACE1 levels^57^. A network analysis based on 407 known substrates of SIRT3 has proposed a novel mechanism for SIRT3-induced BACE1 inhibition through SIRT3, LKB1, AMPK, CREB, PGC-1a, PPARG and BACE1^58^. These findings suggest that SIRT3 activation can decrease Aβ generation by multiple mechanisms and explain the exacerbated amyloid plaque deposition observed in APP/PS1/Sirt3^-/-^ mouse brain.

IDE plays a physiological role in insulin homeostasis via hepatic clearance. Mutations in IDE gene leads to glucose intolerance and hyperinsulinemia^24^. Goto-Kakizaki rat model for T2D is characterized by altered insulin degradation due to mutations in IDE gene^59^. An increase in plasma IDE activity has been reported in both types of diabetes. Constitutive insulin secretion is observed in IDE-KO mice, suggesting that IDE is required for the regulation of glucose-stimulated insulin secretion^60^. Inhibition of IDE activity with a small molecule, was shown to result in glucose intolerance suggesting a physiological role for IDE in insulin clearance^61^. Studies with IDE-KO model suggests that diverse physiological processes are being regulated by IDE, especially in the light of its non-catalytic function. Carlos et al. have suggested complex nature of transcriptional and post-transcriptional regulation of IDE by fasting and feeding^62^ Furthermore, the ubiquitous expression of IDE with a large number of substrates suggests that IDE may not be an ideal therapeutic target.

The findings of this study suggest that SIRT3 is a potential therapeutic target for the treatment of AD. Sirtuins, including SIRT3, require NAD+ as a cosubstrate^63,64^. Administration of NR, a precursor of NAD+, leads to increase in the cellular level of NAD+^65^. In this study, we demonstrate that NR increases the levels of IDE as well as neprilysin and decreases the levels of BACE1 in vivo (Fig. 6). Interestingly, NR also increases the levels of SIRT3, probably by autoregulation. NR has been reported to reverse insulin resistance and glucose intolerance in a mouse model for type 2 diabetes^66^. NR has been shown to be effective in reducing neuroinflammation and improve cognition in Alzheimer’s mouse models^65,67^. Therefore, NR-based treatment can be considered for the treatment of AD with comorbidities.

## Materials and methods

### Animals and treatment

Animal care and all related animal experimental procedures (protocol # 2MR1604M) received prior approval by the Institutional Animal Care and Use Committee at the Rocky Mountain Regional VA Medical Center, Aurora CO. All the animal procedures were performed in accordance with the RMRVAMC animal house facility and followed the guidelines described by the ARRIVE. Mice, 129 Sv (Wild type, stock # 002448), global Sirt3^-/-^ in 129 Sv genetic background (stock # 012755), C57BL/6 (wild type; WT) and APP/PS1 (AD mice, stock # 5864) in C57BL/6 genetic background, were obtained from Jackson Laboratory (Bar Harbor, ME). Global Sirt3^-/-^ mice in C57BL/6 background were provided by Louise Lantier, Vanderbilt University Medical Center (Nashville, TN). Comorbid AD (APP/PS1/Sirt3^-/-^) mice were generated as previously described^7^. The brain sample from this study was used for RNA-seq analysis. Western diet feeding of wild type, Sirt3^-/-^^8^ and APP/PS1^15^ mice were as previously described. The blood and brain samples from these previous studies were used to determine the IDE levels in the current study.

### NR treatment

Seven-months old C57BL/6 male mice (WT, 6/group) were switched to AIN-93G pellet diet (TD.94045, ENVIGO, Madison, WI) or 0.4% nicotinamide riboside (NR, Cat # 00014315, ChromaDex, Irvine, CA) in AIN-93G pellet diet for 3 months. At the termination of the study, mice were subjected to CO2 asphyxiation followed by euthanasia. Blood was collected by cardiac puncture in BD Microtainer tubes coated with K2 EDTA followed by centrifugation at 4 °C, and separated plasma samples were stored at − 80 °C for ELISA analysis. The brain was snap frozen in liquid nitrogen and stored at − 80 °C for protein analyses.

### Cell culture studies

Mouse microglial cell line (BV2) was provided by Dr. Dennis Selkoe (Harvard Medical School, Boston, MA). BV2 cells were grown at 37 °C and 5% CO_2_ in an advanced DMEM/F12 medium (Cat # 12634-010, Gibco, Gaithersburg, MD) containing 10% fetal bovine serum and 1% penicillin–streptomycin. Sirt3 was silenced by 70% in this cell line using Sirt3 shRNA, as previously described^15^. Briefly, cells were infected with Lentiviral particles, Sirt3 shRNA (Cat # sc 61,556) or control shRNA (Cat # sc108080), using polybrene (0.5 µg/ml; Cat # sc134220). Puromycin dihydrochloride (8 µg/ml, Cat # sc10807), in DMEM/F12 media supplemented with 10% FBS, was used to select the infected cells. The infected cells were pooled and maintained in the same medium.

### Treatment

Cells were grown to 65–75% confluence, and treated with different concentration of NR (0.5, 1.0 and 2.0 mM) for 24 and 48 h. Treated cells were harvested and total cell lysates were prepared in mammalian protein extraction buffer (Pierce, Rockford, IL) supplemented with complete protease inhibitor cocktail (Cat # 11836153001, Millipore Sigma Burlington, MA).

### ELISA

Plasma IDE levels were measured using a colorimetric kit (Cat. # MBS2884110, MyBioSource, San Diego, CA). The absorbance was measured spectrophotometrically at 450 nm using a BioTek Synergy H1 microplate reader (Winnoski, VT). The IDE concentration (ng/ml) was determined and expressed as percent of control.

### Western blot analysis

Total protein extracts from mouse brain frontal cortical tissue samples and cultured cells were analyzed by western blot analysis as previously described^15^. Briefly, samples with equal amount of protein (25–40 µg) were run on a gradient 4–20%, 8% or 12% SDS-PAGE ( BIO-RAD, Hercules, CA) and transferred to polyvinylidene difluoride (PVDF) membrane. The membranes were incubated in TBST containing 5% non-fat milk for 1 h at RT to block non-specific binding. Subsequently, membranes were incubated with primary antibodies [SIRT3 (Cat # 5490), BACE1 (Cat # 5606) and β actin (Cat # 4967) from Cell Signaling Technology, Danvers, MA; Antibodies for Insulin-degrading enzyme (Cat # ab32216) and neprilysin (Cat # ab227659) were purchased from AbCam, Cambridge, MA] overnight on a shaker at 4 °C. The following day, membranes were washed three times with TBST, and then incubated with alkaline phosphatase conjugated appropriate secondary antibody for 1 h at RT. After an additional three washes in TBST, the signals were visualized by CDP-Star reagent (Sigma Aldrich-St Louis, MO), using ChemiDoc Imaging System and Image Lab touch software (BIO-RAD, Hercules, CA). As edges of the blots are not clearly visible or very dim in grey backgrounds, composite color tiff images are added as a supplementary Figure. In these images, protein standards, and target proteins are seen in blue and blot edges were clearly visible. Some blots were cropped/multiplexed when proteins of interest are significantly separated in molecular weights. After image capture, blots were stripped and re-probed for β-actin content to ensure equal protein loading. The band intensities were quantified with reference to β-actin control bands using Adobe Photoshop 6.0 software (Adobe systems, San Jose, CA).

### Assay of IDE activity

Activity of IDE was measured as reported previously^68^ using fluorometric SensoLyte® 520 IDE activity assay kit (Cat # AS-72231, AnaSpec, Inc, Fremont, CA, USA) following the manufacturer’s protocol. Briefly, brain frontal cortical tissue extracts were homogenized in cold assay buffer (AnaSpec, Inc, Fremont, CA, USA) supplemented with complete protease inhibitor cocktail tablet (Cat # 11836170001, Sigma Aldrich) plus 0.3 mM PMSF. Homogenates were kept on ice for 30 min, followed by centrifugation at 10,000 X g for 15 min at 4 °C. Protein concentration was measured in the supernatants. Subsequently, enzyme reaction was set up by adding 50 µl of tissue lysates containing 100 µg protein/well in a 96-well black opaque plate. The enzymatic reaction was started by adding 50 µl fluorogenic substrate into each well. The plate was shaken for 30 s, and the reaction was incubated at 37 °C for 60 min in the dark. As a positive control, purified recombinant human IDE provided in the kit was used. The fluorescence intensity was measured at Ex/Em = 490 nm/520 nm using GloMax plate reader (Promega, Madison WI). The fluorescence readings from the wells containing the assay buffer without tissue lysates were used as a background fluorescence. The background reading was subtracted from the reading of the samples, and results are expressed as fluorescence intensity/µg protein.

### RNA isolation, QC and library construction

Total RNA was isolated from brain cortical samples. The quality of RNA was checked, using the Agilent 2100 Bioanalyzer system (Santa Clara, CA) with a RIN cutoff of 7. A total of 2 µg of RNA per sample was used as input material for the generation of sequencing libraries using NEBNextULtra RNA Library Prep Kit from Illumina. Briefly, mRNA was purified from total RNA, using oligo (dt) magnetic beads, and sheared into short fragments of ~ 200 bases. These fragmented mRNAs were used as templates for cDNA synthesis, and the cDNAs was PCR-amplified for the library preparation. The cDNA libraries were sequenced (25 million reads), using Illumina NexSeq550 platform at Novogene (Chula Vista, CA). Following the removal of low-quality reads and adaptor sequences from the raw reads, clean reads were obtained and mapped to the mouse genome assembly (GRCm38/mm10), using TopHat software, allowing up to two base mismatches. The gene expression levels were calculated, using the reads per kilo bases per million reads (RPKM) method.

### Differentially expressed genes (DEG), GO and KEGG analyses

The differential gene expression analysis was performed using DESeq2 package. Benjamini and Hochberg’s approach was used for controlling the false discovery rate. The genes with an adjusted *P* value of < 0.05 were assigned as differentially expressed. After generating the volcano plots for comparison between groups, DEG lists were submitted to the databases of Gene ontology (GO) and Kyoto Encyclopedia of Genes and Genomes (KEGG) for enrichment analysis^69^. For the functional annotation of differentially expressed genes, the web-based DAVID Bioinformatics Resources v 6.8 tool was used (https://david.ncifcrf.gov).

### Statistical analysis

GraphPad Prism (Version 9.0.2, GraphPad Software Inc, San Diego, CA, USA) and SigmaStat (Version 3.5, Jandel Scientific Software, San Jose, CA, USA) were used for statistical analysis. Statistical significance between two groups were determined using unpaired t-test, while one way ANOVA followed by Tukey post-hoc analysis was performed for multiple groups comparison. Two-sided *P* values of ≤ 0.05 were stated as statistically significant.

## Supplementary Information

Supplementary Information.

## Data Availability

The datasets generated in this study are available at the GEO repository (https://www.ncbi.nlm.nih.gov/geo/) and the accession number is GSE216081.
