# Supplementary material for: Sirt3 deficiency induced down regulation of insulin degrading enzyme in comorbid Alzheimer’s disease with metabolic syndrome
Source: Sci Rep. 2022 Nov 17;12:19808. doi: 10.1038/s41598-022-23652-5 (PMC9672095; doi:10.1038/s41598-022-23652-5)
Supplement: Supplementary file 1 — Supplementary Information. [file 41598_2022_23652_MOESM1_ESM.pdf]

SIRT3

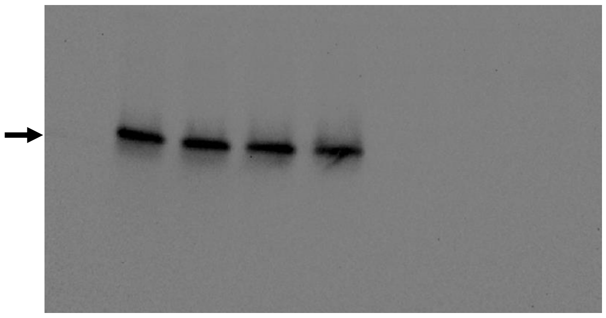

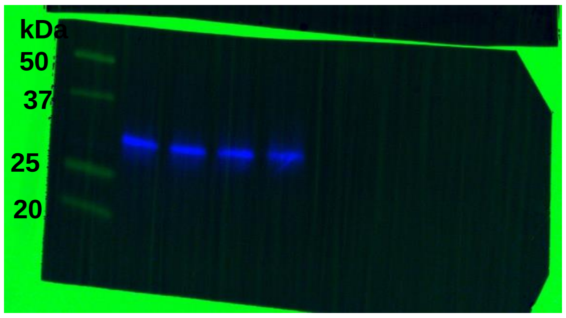

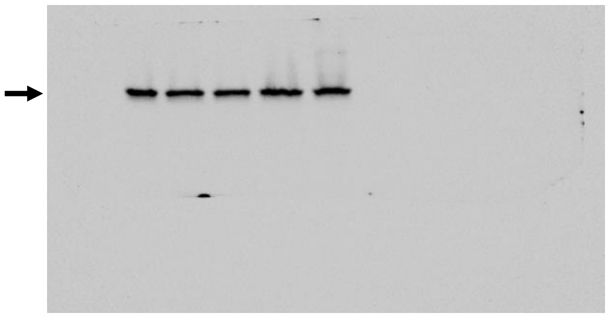

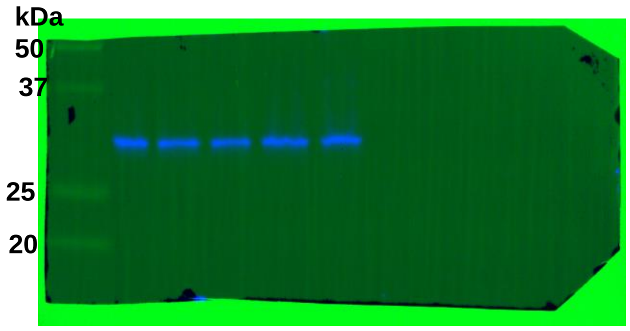

IDE

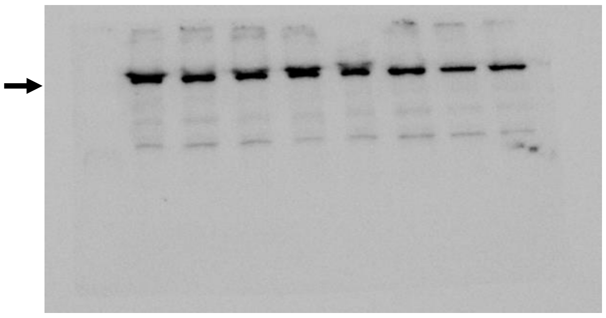

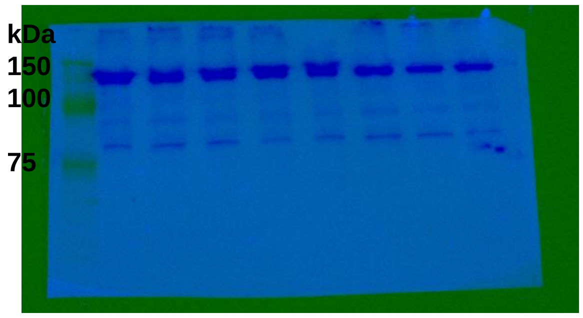

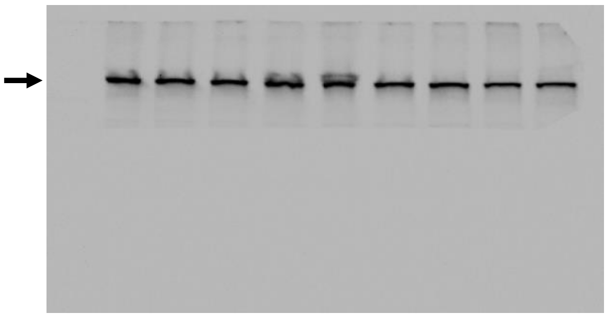

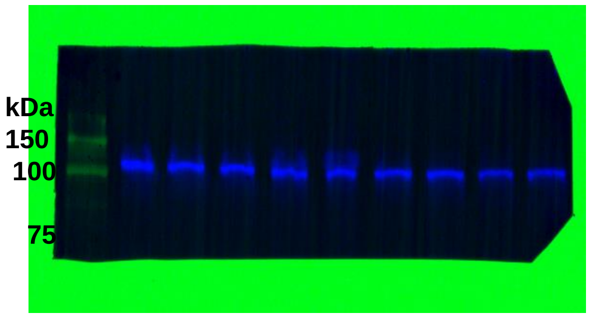

β-actin

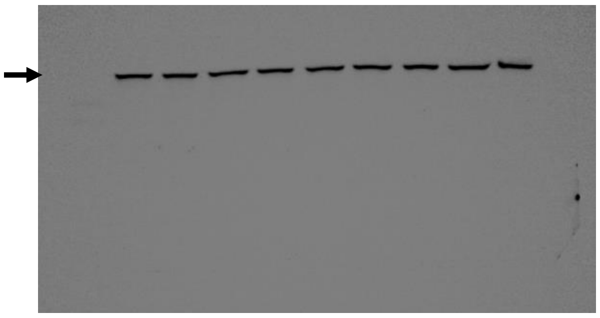

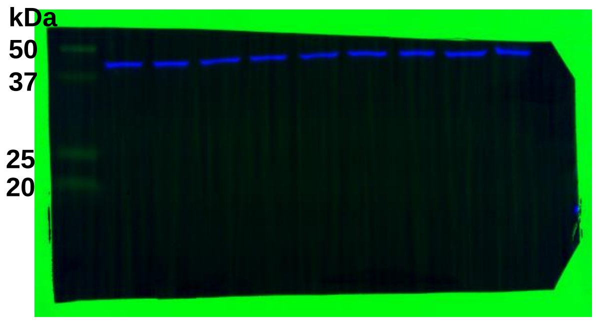

Figure 5A- Full blot replicates

SIRT3

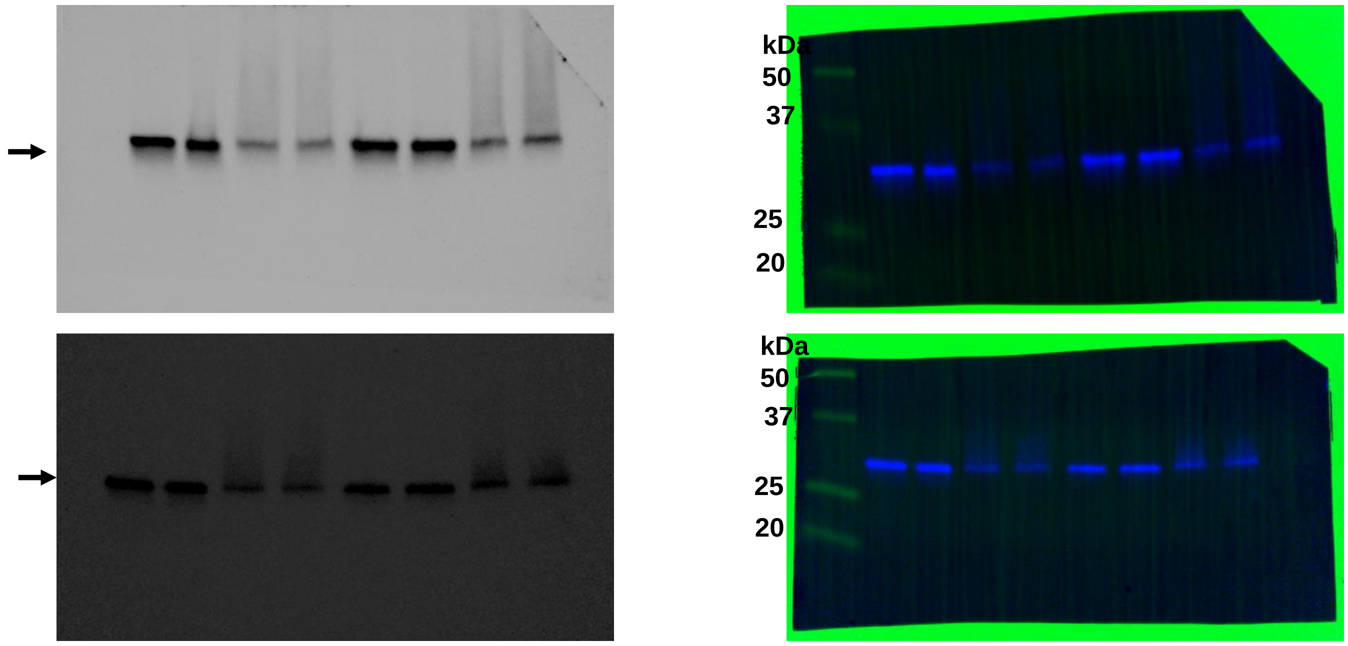

IDE

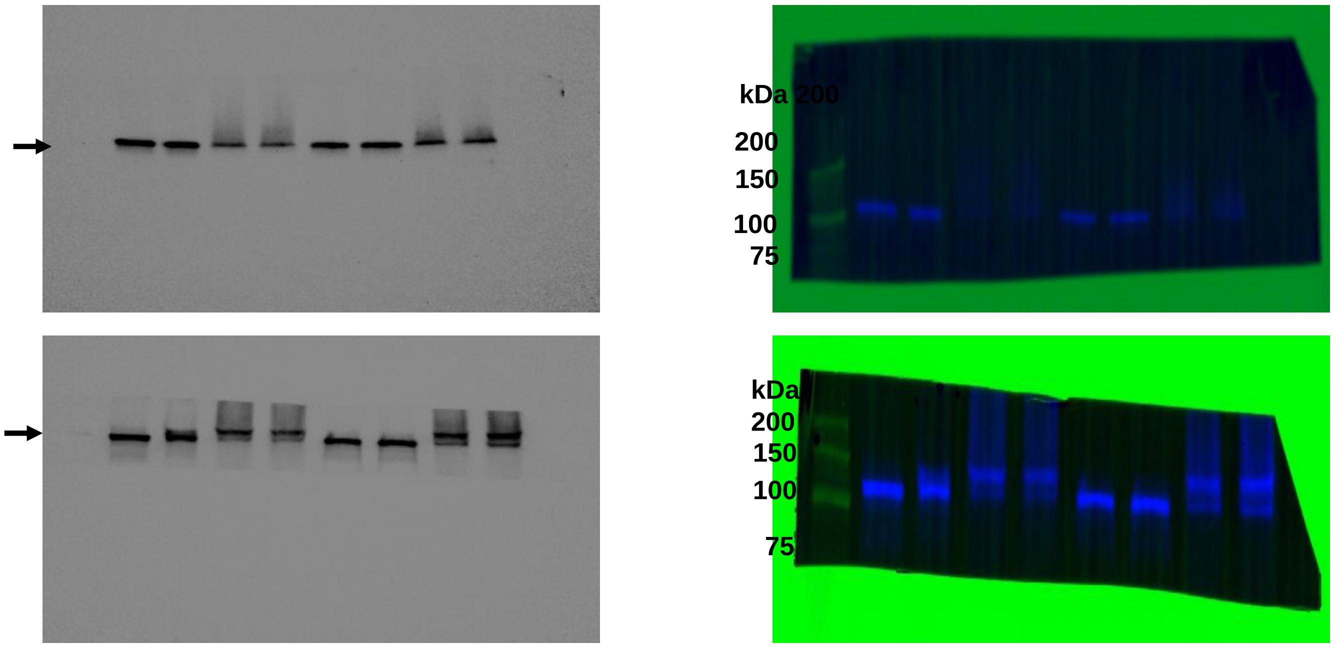

β-actin

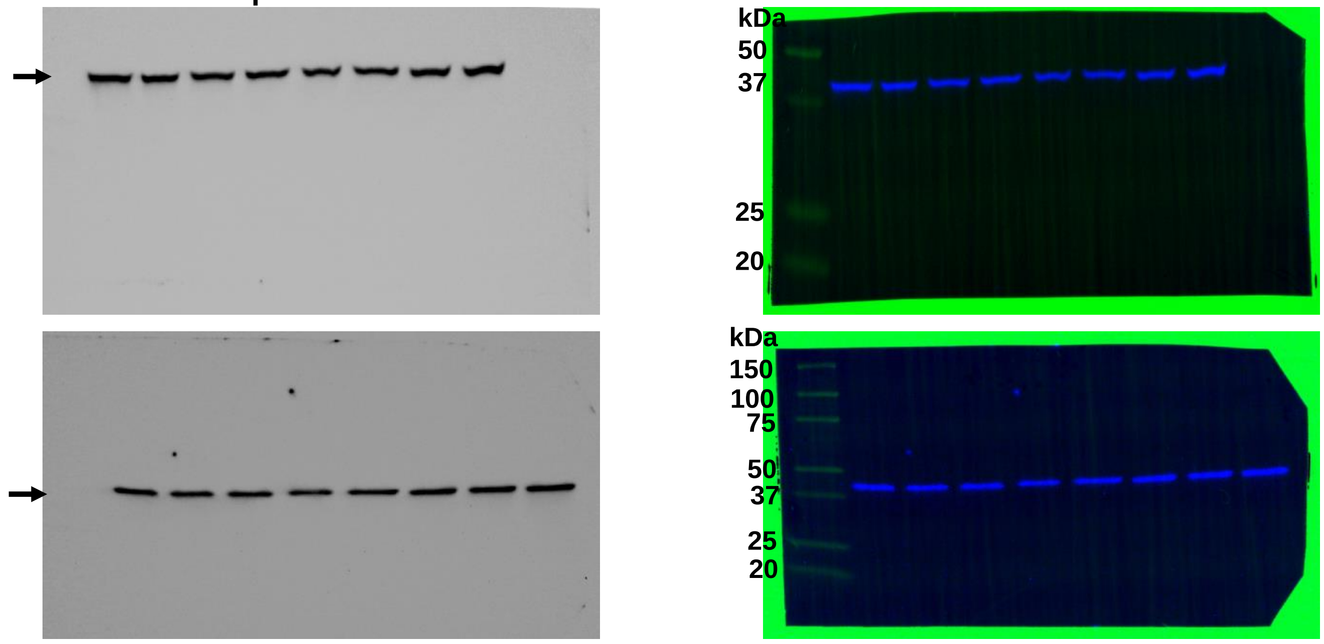

Figure 5B- Full blot replicates

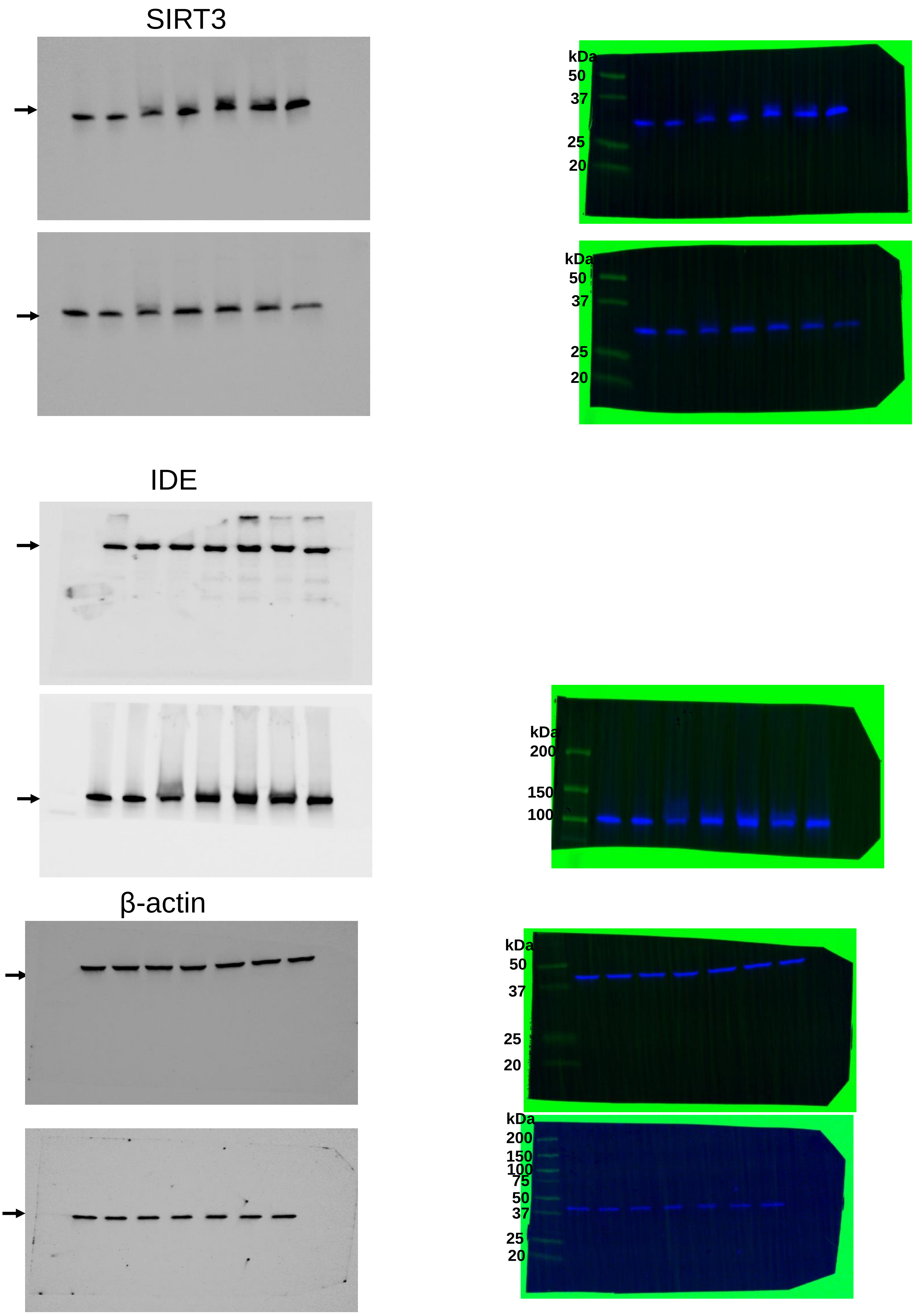

Figure 6A- Full blot replicates

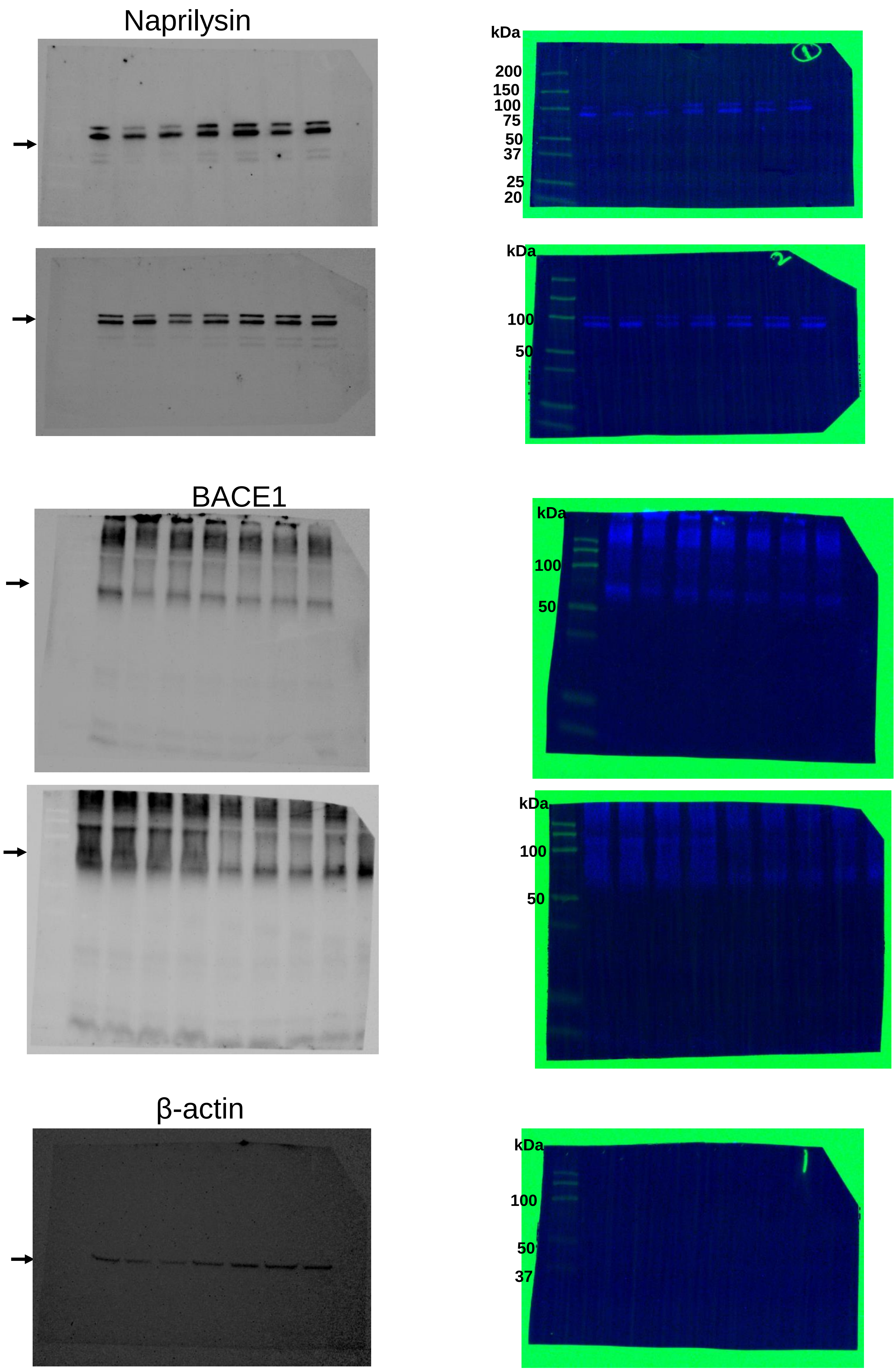

Figure 6B- Full blots

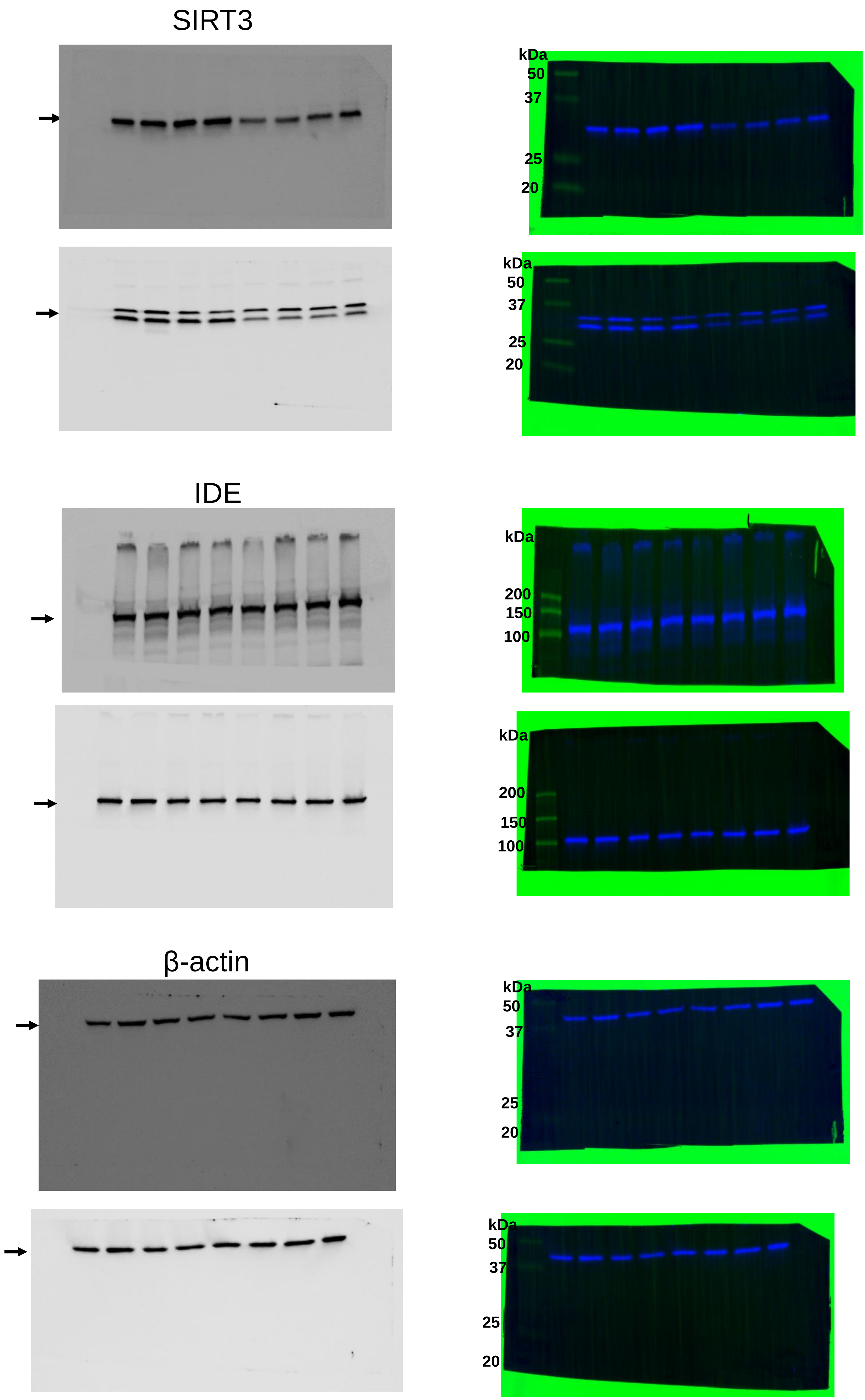

Figure 7A- Full blot replicates

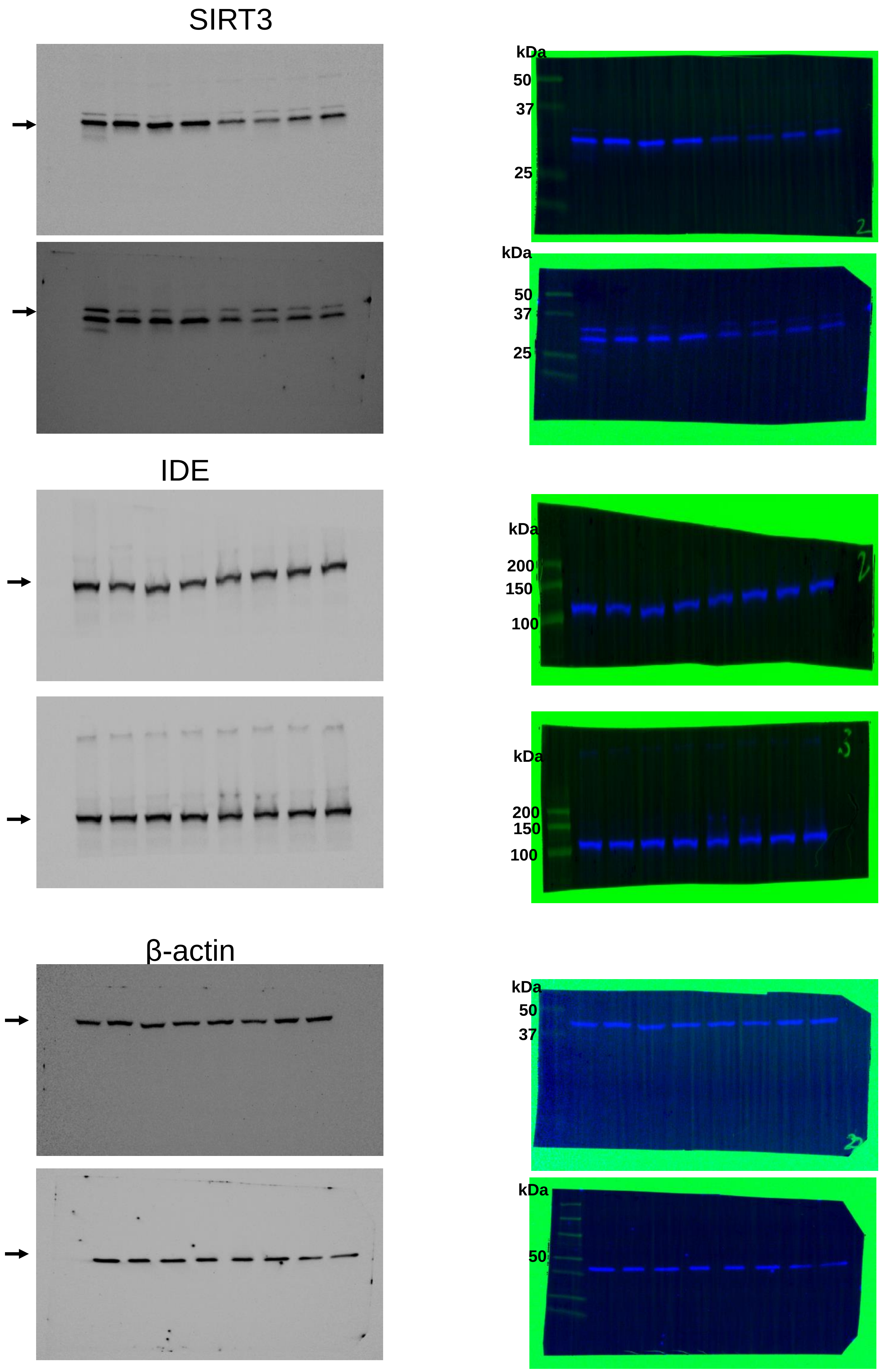

Figure 7B- Full blot replicates
